# Mean platelet volume-to-lymphocyte ratio: a novel biomarker associated with overall survival in patients with nonmetastatic clear cell renal cell carcinoma treated with nephrectomy

**DOI:** 10.1007/s11255-020-02379-0

**Published:** 2020-01-17

**Authors:** Marcin Życzkowski, Zbigniew Kaletka, Pawel Rajwa, Grzegorz Rempega, Paweł Stelmach, Rafał Bogacki, Olga Łach-Wojnarowicz, Ewa Paradysz

**Affiliations:** 1 Department of Urology, School of Medicine With the Division of Dentistry in Zabrze, Medical University of Silesia in Katowice, 3 Maja Street 13-15, 41-800 Zabrze, Poland; 2 Student Scientific Society, Department of Urology, Medical University of Silesia, 3 Maja Street 13-15, 41-800 Zabrze, Poland

**Keywords:** Renal cell cancer, Renal cell carcinoma, RCC, MPVLR, MPV

## Abstract

**Introduction:**

Renal cell carcinoma is a highly aggressive malignancy that causes significant morbidity and mortality. The rising number of newly diagnosed renal tumors results in a great need to search for new preoperative markers to evaluate the course of the disease and to help select patients who would benefit the most from additional postoperative care. The aim of our study was to evaluate the prognostic value of mean platelet volume-to-lymphocyte ratio (MPVLR) in patients undergoing nephrectomy for nonmetastatic clear cell renal cell carcinoma (ccRCC).

**Materials and methods:**

A total number of 344 patients with proven nonmetastatic ccRCC treated with radical or partial nephrectomy at our institution between January 2003 and December 2012 were included in our analysis. Based on the optimal cut-off value of MPVLR, which was determined by the receiver operating characteristic curve, our study population was divided into two groups, with low and high MPVLR. Differences in overall survival between groups were compared using the Kaplan–Meier method with log-rank testing. The Cox proportional hazards regression model was applied to perform univariate and multivariate analysis.

**Results:**

Study subjects with high MPVLR were older and had more advanced tumors. Tumor necrosis and higher TNM stages were also more prevalent in this group of patients. Mortality in patients with high MPVLR was significantly higher than in patients with low MPVLR. In the multivariate analysis, after adjustment for pathological and clinical covariates, high MPVLR (≥ 3.61) was independently associated with higher long-term overall mortality in nonmetastatic ccRCC patients.

**Conclusion:**

MPVLR is an easily obtainable prognostic marker for overall survival in nonmetastatic ccRCC patients treated with nephrectomy.

## Introduction

Renal cell carcinoma (RCC) is a highly aggressive malignancy that is one of the most common malignancies worldwide [1, 2]. It represents 2–3% of all cancers in global population [1]. Men are almost twice more likely to develop RCC than women, with peak incidence occurring between 60 and 70 years of age [1]. The most common risk factors are inter alia: cigarette smoking, obesity, hypertension and diabetes mellitus, which are considered as civilization diseases [1, 2]. Clear cell RCC (ccRCC), which is the main interest of our paper, is the most common histologic type of kidney cancer [1, 3]. It is responsible for approximately 65–75% of RCC cases [3]. The first-line treatment for localized kidney cancer is surgical resection, either by partial or radical nephrectomy [1]. Nevertheless, approximately 10–50% of patients undergoing surgical treatment develop a disease recurrence after surgical treatment, which is associated with higher long-term mortality [4–6]. Nowadays, due to significant improvement in diagnostic radiology, we observe rising prevalence of incidental renal tumors [2, 7]. Despite the fact that the 5-year overall survival (OS) for all types of RCC has increased in recent years, it still remains unacceptable [2]. In addition, the rising number of newly diagnosed small renal masses, especially in patients with many comorbidities, results in a great need to search for new preoperative markers to evaluate the course of the disease and to help select patients who would benefit the most from additional postoperative care [1, 8, 9]. One of the recently implemented complete blood count (CBC)-based indexes is mean platelet volume-to-lymphocyte ratio (MPVLR). It was first introduced in 2016 by Hudzik et al. as a potential prognostic marker in patients with diabetes mellitus and myocardial infarction [10]. Regarding the components of the MPVLR and the existing crosstalk between blood cells and cancer, we have assumed that MPVLR might have a prognostic value in surgically treated nonmetastatic ccRCC patients. Hereby, we present the first study which confirms the association between preoperative MPVLR and OS of M0 ccRCC patients undergoing partial or radical nephrectomy.

The first aim of our study was to evaluate the prognostic value of MPVLR in patients who underwent partial or radical nephrectomy for nonmetastatic ccRCC. The second goal was to analyze MPVLR in comparison with other clinical and pathological features.

## Materials and methods

### Patients

Medical records of 344 consecutive patients who underwent partial or radical nephrectomy for M0 clear cell renal cell carcinoma (ccRCC) at our institution between January 2003 and December 2012 were retrospectively analyzed. Histopathological, demographic and laboratory data, information on comorbidities and operative methods were retrieved from pathology reports from the Department of Pathology and from medical reports from the Department of Urology at our institution. Patients suffering from non-ccRCC tumor, distant metastases and those with confounding severe inflammatory and neoplastic processes were initially excluded from the study. Due to the change in tumor staging system during the observational period, TNM classification was assessed in compliance with the American Joint Committee on Cancer, 7th edition (2010). Tumor grading was performed according to the Fuhrman system. Mean platelet volume (MPV) and lymphocyte count were obtained from preoperative complete blood counts which were performed median 3 (range 0–26) days prior to nephrectomy, and were available for all analyzed patients. Mean platelet volume-to-lymphocyte ratio (MPVLR) was calculated as MPV divided by lymphocyte count (× 10^3^/mm^3^).

### Patient follow-up

Information on survival was obtained from the Centre for Document Personalization of the Polish Ministry of Interior and Administration, which stores, among others, the exact date of death of every Polish citizen since the mid-1980s. Complete follow-up data were available for all analyzed patients. The primary study endpoint was OS, defined as time (months) from surgical intervention to all-cause death or the date of the end of follow-up, which was May 15, 2016.

### Statistical analysis

Continuous variables are presented as median (interquartile range). Dichotomous variables are presented as percentages. Comparisons of baseline characteristics, laboratory and pathological findings were performed using the Mann–Whitney *U* test and the Chi-squared test for continuous and categorical variables, respectively. Optimal cut-off value of MPVLR for overall mortality prediction was determined using receiver operating characteristic (ROC) curve analysis and the Youden method. Based on optimal cut-off value of MPVLR, study population was divided into two groups: high MPVLR group with MPVLR higher than or equal to the optimal cut-off value, and low MPVLR group with MPVLR lower than the optimal cut-off value. Differences in OS between groups were compared using the Kaplan–Meier method with log-rank testing. The Cox proportional hazards regression model was applied to perform univariate and multivariate analysis. Variables that had *p* value lower than 0.05 in univariate analysis were entered into multivariate analysis. Statistical analyses were performed using the STATISTICA 12 software with Medical Bundle (StatSoft Inc., Tulsa, Oklahoma, USA).

## Results

**Fig. 1:**
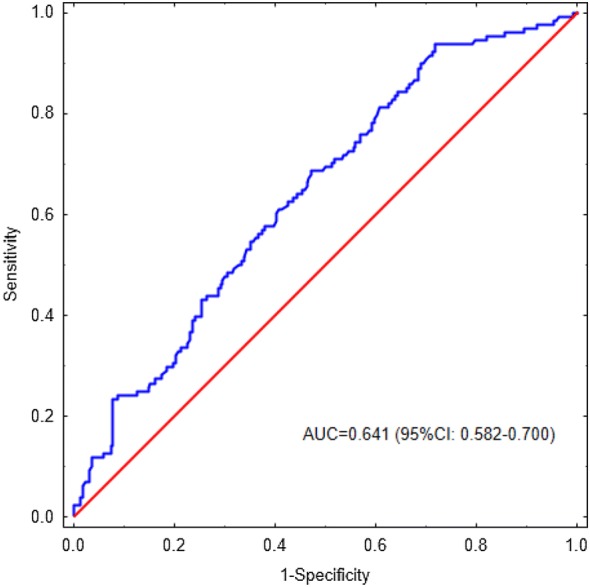
Receiver operating characteristic (ROC) curve for MPVLR in the prediction of overall mortality in patients with nonmetastatic CCRCC undergoing nephrectomy. *AUC* area under the curve, *CI* confidence interval, *MPVLR* mean platelet volume-to-lymphocyte ratio, *CCRCC* clear cell renal cell carcinoma

**Fig. 2:**
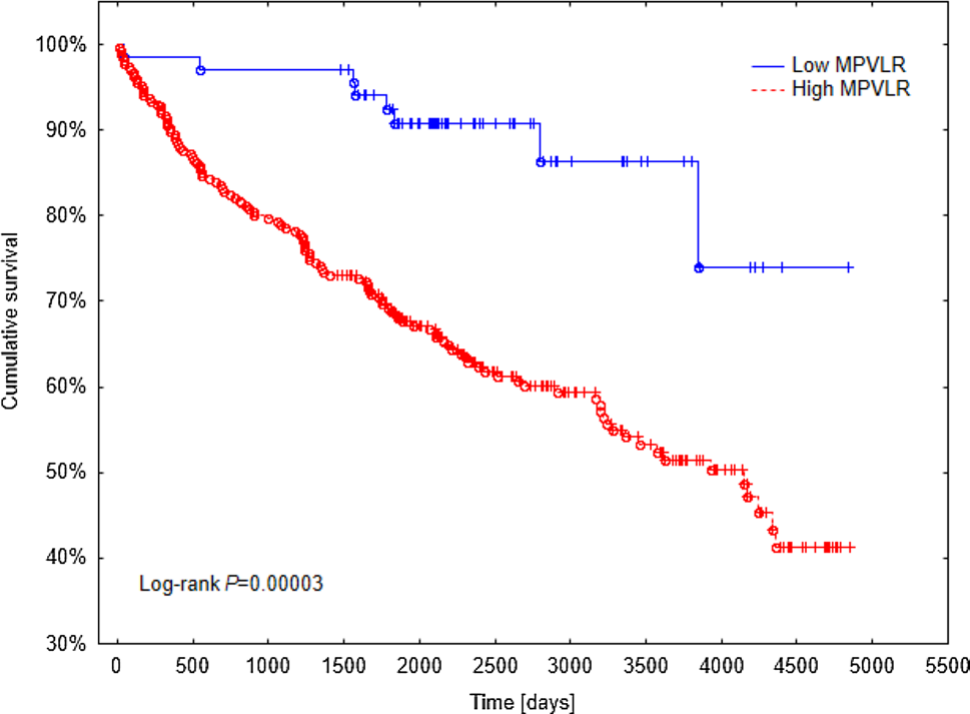
The Kaplan–Meier curves for overall survival of ccRCC patients with high and low MPVLR

Median follow-up was 2184 (interquartile range 1553–3243) days. During the observation period, overall survival rate in the whole study population was 62.8%. ROC curve for MPVLR in the prediction of overall mortality is depicted in Fig. 1. Optimal cut-off value of MPVLR for death prediction was 3.61. Comparison of baseline characteristics, laboratory and pathological findings between low and high MPVLR groups is presented in Table 1. Subjects with high MPVLR were older and had more pathologically advanced tumors. Tumor necrosis and higher TNM stages were also more prevalent in this group of patients. There were no differences between patients with high and low MPVLR in terms of sex, lymph node involvement, presence of sarcomatoid feature, tumor grade and tumor size. Mortality in patients with high MPVLR was significantly higher than in ones with low MPVLR (Fig. 2). In multivariate analysis, after adjustment for clinicopathological covariates, high MPVLR (≥ 3.61) was independently associated with greater long-term mortality in nonmetastatic ccRCC patients (Table 2).

**Table 1:** Baseline characteristics, laboratory and pathological findings

	Total *n* = 344	MPVLR		
		Low (< 3.61) *n* = 69	High (≥ 3.61) *n* = 275	*p* value
Sex				0.07
Male	50.29%	40.58%	52.73%	
Female	49.71%	59.42%	47.27%	
Age (years)	63.0 [54.0–70.0]	56.0 [52.0–64.0]	65.0 [56.0–71.0]	0.00007
BMI [kg/m^2^]	27.17 [24.57–30.67]	28.87 [24.85–32.05]	27.1 [24.51–30.17]	0.19
Hemoglobin (g/dl)	13.8 [12.7–14.8]	13.8 [13.1–14.7]	13.7 [12.4–14.9]	0.35
Red blood cell count (10^6^/mm^3^)	4.54 [4.28–4.87]	4.64 [4.38–4.92]	4.51 [4.26–4.87]	0.13
White blood cell count (10^3^/mm^3^)	6.7 [5.6–8.2]	8.4 [6.6–9.1]	6.35 [5.5–7.69]	< 0.0001
Lymphocytes (10^3^/mm^3^)	1.9 [1.5–2.4]	2.7 [2.4–3.2]	1.8 [1.5–2.1]	< 0.0001
Neutrophils (10^3^/mm^3^)	3.85 [3.1–5.1]	4.1 [3.2–5.4]	3.8 [3.0–4.9]	0.07
Monocytes (10^3^/mm^3^)	0.4 [0.32–0.6]	0.5 [0.4–0.6]	0.4 [0.3–0.54]	0.06
Platelets (10^3^/mm^3^)	238.0 [194.0–297.0]	276.0 [231.0–321.0]	230.0 [187.0–287.0]	0.0007
MPV (fl)	9.4 [8.3–10.8]	8.3 [7.5–9.3]	9.75 [8.4–11.0]	< 0.0001
MPVLR	4.95 [3.79–6.27]	3.21 [2.72–3.38]	5.43 [4.5–6.58]	< 0.0001
Sarcomatoid feature				0.85
Present	1.83%	1.54%	1.90%	
Absent	98.17%	98.46%	98.10%	
pT stage				0.01
pT1	64.97%	80.00%	61.34%	
pT2	12.87%	12.31%	13.01%	
pT3	21.56%	7.69%	24.91%	
pT4	0.60%	0.00%	0.74%	
Lymph node involvement				0.66
pN0	96.51%	95.38%	96.73%	
pN1	3.49%	4.62%	3.27%	
TNM stage				0.05
I	64.07%	78.46%	60.59%	
II	12.28%	9.23%	13.01%	
III	23.05%	12.31%	25.65%	
IV	0.60%	0.00%	0.74%	
Tumor grade				0.23
G1	21.62%	20.00%	22.01%	
G2	48.35%	58.46%	45.90%	
G3	24.02%	15.38%	26.12%	
G4	6.01%	6.15%	5.97%	
Tumor size (mm)	50.0 [35.0–70.0]	50.0 [30.0–65.0]	50.0 [35.0–70.0]	0.28
Nephrectomy				0.4
Partial	33.43%	37.68%	32.36%	
Radical	66.57%	62.32%	67.64%	
Tumor necrosis				0.01
Present	14.51%	4.62%	17.06%	
Absent	85.49%	95.38%	82.94%	
Overall survival	62.79%	88.41%	56.36%	0.00003^a^

Continuous variables are presented as median [interquartile range]. Dichotomous variables are presented as percentages

*CCRCC* clear cell renal cell carcinoma, *MPV* mean platelet volume, *MPVLR* mean platelet volume-to-lymphocyte ratio

^a^Log-rank

**Table 2:** Predictors of overall mortality in patients with nonmetastatic CCRCC treated with nephrectomy—univariate and multivariate analysis

	Univariate analysis		Multivariate analysis	
	HR (95% CI)	*p* value	HR (95% CI)	*p* value
Sex		0.70		
Female	Reference			
Male	1.07 (0.76–1.51)			
Age		< 0.0001		0.0004
< 65 years	Reference		Reference	
≥ 65 years	2.43 (1.69–3.47)		2.01 (1.37–2.97)	
pT stage		< 0.0001		< 0.0001
pT1 + pT2	Reference		Reference	
pT3 + pT4	4.33 (3.04–6.17)		3.08 (1.95–4.88)	
Lymph node involvement		0.01		0.74
pN0	Reference		Reference	
pN1	2.41 (1.22–4.74)		0.87 (0.38–1.98)	
Nephrectomy		0.003		0.57
Radical	Reference		Reference	
Partial	0.54 (0.36–0.81)		1.15 (0.71–1.86)	
Sarcomatoid feature		0.03		0.38
Absent	Reference		Reference	
Present	3.09 (1.14–8.39)		0.58 (0.17–1.96)	
Tumor necrosis		< 0.0001		0.0002
Absent	Reference		Reference	
Present	4.36 (2.91–6.53)		2.45 (1.53–3.91)	
Grading		< 0.0001		0.003
G1 + G2	Reference		Reference	
G3 + G4	2.73 (1.92–3.86)		1.92 (1.24–2.95)	
MPVLR		0.0001		0.04
Low (< 3.61)	Reference		Reference	
High (≥ 3.61)	4.01 (1.96–8.21)		2.2 (1.05–4.6)	

*CCRCC* clear cell renal cell carcinoma, *HR* hazard ratio, *CI* confidence interval, *MPVLR* mean platelet volume to lymphocyte ratio

## Discussion

This study addressed the association of MPVLR with overall survival in study subjects with nonmetastatic ccRCC undergoing surgical treatment. Hereby, we present the first clinical study, which confirms a significant, prognostic relationship between high preoperative MPVLR and greater long-term mortality of cancer patients.

Up to now, numerous studies reported different prognostic factors affecting OS in RCC patients, but none of them emerged as a perfect indicator for prognosis [1, 11, 12]. The paradox lies in the fact that a new preoperative biomarker is needed to identify patients with highest long-term mortality, as such patients would benefit most from additional treatment; yet, the majority of currently reported predictors are based on pathologic examination of postoperative specimen, such as tumor grade and stage, cellular anaplasia and tumor histology [1]. The prognostic value of these models is considered to be insufficient and thus needs further improvements, which could be obtained with the addition of a preoperative biomarkers. However, it must be pointed out that the biggest drawbacks of most of the currently studied markers, such as hypoxia-inducible factor (HIF) or microRNAs (miRNAs,) are their high cost and low availability [13–15]. The solution might be found in cheap and easy obtainable blood-based indicators. Different complete blood count (CBC) parameters were reported to have an association with overall survival in RCC, such as neutrophil count, platelet count, neutrophil-to-lymphocyte ratio and mean platelet volume [16–21]. These markers are routinely used in everyday practice. Mean platelet volume-to-lymphocyte ratio (MPVLR) was reported to be associated with coronary collateral circulation development in patients with stable angina pectoris as well as with poor short- and long-term prognosis in patients with diabetes mellitus and acute myocardial infarction [10, 22]. To our knowledge, this is the first study to investigate the association between MPVLR and overall survival in cancer patients.

MPVLR value, which is counted as MPV divided by lymphocyte count, represents the interplay between cancer and two different blood cells lines. Both, platelets and lymphocytes, take part in carcinogenesis and have separate developmental pathways, that are influenced by growing tumor. We hypothesize that elevated MPVLR might reflect a deteriorated interaction between tumor cells, blood platelets and lymphocyte-dependent immune response. A growing number of studies deliver evidence to support the potentially important role of platelets in the process of tumor cell growth and metastasis [23, 24]. There is strong evidence, that the actual feature that illustrates more precisely the function and activity of platelets is their size—MPV [25]. Larger platelets contain more dense granules and have a higher thrombogenocity potential [26]. Considering MPV prognostic value in RCC patients, Seles et al*.* retrospectively evaluated 652 patients with RCC and concluded that MPV is a significant predictor of recurrences and cancer-specific deaths [20]. Also, Yun et al*.* determined the association between lower mean platelet volume and prognosis in RCC, and reported that low MPV is a predictor of adverse clinical outcome [19]. On the other hand, decreased level of lymphocytes is strongly associated with the suppression of the immune system during carcinogenesis [27]. Saroha et al. retrospectively analyzed preoperative blood cell counts in 430 patients with ccRCC undergoing primary surgical treatment [27]. The study showed that lymphopenia was associated with lower overall survival regardless of tumor grade and stage, nuclear grade, patient’s age, tobacco smoking and comorbidity index [27]. As described, the relationship between RCC and MPVLR remains complex, as MPVLR may reflect the aggressiveness of the tumor, altered platelet-mediated immunity and decreased lymphocyte-mediated immune response.

We acknowledge that the retrospective nature of our study and the fact that we were not able to obtain postoperative therapy data and information on comorbidity burden of analyzed population, as well as relatively small sample size are major limitations of our research. Due to the fact that the RCC is most often diagnosed in elderly patients, further studies are needed to validate the prognostic value of MPVLR in younger cohorts. Due to aforementioned limitations, our finding should be considered as preliminary.

## Conclusions

We have to report several key conclusions of our study. To our knowledge, this is the first study, which confirms the association between MPVLR and OS in patients with nonmetastatic ccRCC treated surgically. In our opinion, MPVLR may become a useful preoperative marker in everyday urological practice as it allows for a quick selection of patients with a higher risk of overall mortality in long-term observation. It must be also emphasized that MPVLR was significantly associated with TNM staging components and tumor necrosis, which may also be a valuable diagnostic marker in preoperative cancer assessment. To sum up, thanks to this simple, cheap and easily obtainable marker, there may be a chance to distinct patients requiring special postoperative care and to implement a more intensive treatment. Making key decision concerning the course of treatment can, through the use of MPVLR, proceed faster, more efficiently and with greater benefit for the patient. Nonetheless, further studies are necessary to validate the prognostic value of MPVLR in patients with ccRCC treated with nephrectomy.
